# Ultrasound combined with molecular genetics to diagnose hereditary Renpenning syndrome in early pregnancy: a case report

**DOI:** 10.3389/fgene.2025.1575378

**Published:** 2025-07-21

**Authors:** Yongmei Shen, Lei Zhang, Yaqi Li, Liying Yao, Jiasong Cao, Qimei Lin, Maolin Nie, Hefei Wang, Rongxin Wei, Ying Chang

**Affiliations:** ^1^Tianjin Key Laboratory of Human Development and Reproductive Regulation, Tianjin Central Hospital of Gynecology Obstetrics, Tianjin, China; ^2^Tianjin Institute of Obstetrics and Gynecology, Tianjin Central Hospital of Gynecology Obstetrics, Tianjin, China; ^3^Obstetrics and Gynecology, Tianjin Medical University, Tianjin, China; ^4^Medical School, Tianjin University, Tianjin, China

**Keywords:** *PQBP1*, Renpenning syndrome, nuchal translucency, ultrasound, prenatal diagnosis

## Abstract

Renpenning syndrome is a rare X-linked genetic disorder caused by variants in the PQBP1 gene, but the information about its prenatal presentation is very limited. A 35-year-old woman experienced two male pregnancies with thickened nuchal translucency (NT) (5.5 mm and 5 mm). She went to our prenatal diagnosis center for the current natural conception during the second pregnancy. Trio-whole exome sequencing (TrioWES) of chorionic villus biopsy revealed a 666-bp genetic deletion (chrX:48755195-49760422) in the fetus, inherited from the mother, which included *TIMM17B* and *PQBP1*. The couple opted for termination of pregnancy. During the third pregnancy, systematic fetal screening was performed in early pregnancy. An ultrasound examination at 12+1 weeks revealed a thickened NT (6.5 mm), nasal bones abnormalities and a cleft palate. Ultrasound examination at 16 weeks showed ventricular septal defect (VSD), and mild enlargement of the lateral ventricles in the fetus. Chorionic villus biopsy samples were tested for Multiplex Ligation-dependent Probe Amplification (MLPA), showing a 666-bp genetic deletion, inherited from the mother. The couple opted for termination of pregnancy, and the male fetus had a sunken nose and cup-shaped ears leading to a diagnosis of Renpenning syndrome. In conclusion, this emphasized the importance of early systematic pregnancy screening. Increased NT in the first trimester, especially when present in conjunction with ultrasound structural abnormalities such as nasal bone abnormalities, VSD, and mild bilateral ventriculomegaly, emphasized the importance of genetic testing, including chromosome testing, genomic testing, and Whole-exome sequencing.

## Introduction

Renpenning Syndrome is a rare X-linked genetic disorder caused by variants in the *PQBP1* gene. Symptoms of this condition include intellectual disability (HP:0001249), microcephaly (HP:0000252), short stature (HP:0004322), small testes (HP:0008734), and facial dysmorphism (HP:0001999). Studies using different models have shown that PQBP1 plays a crucial role in neural development and function (Cheng et al., 2023). These patients showed moderate intellectual disability, poor autonomy, communication and social interaction disorders, learning difficulties, and obvious autistic behaviors (Redin et al., 2014). An increasing number of articles have documented Renpenning syndrome. For example, a recent Korean case study identified a novel mutation in the *PQBP1*(NM_001032381.2) gene, leading to the diagnosis of Renpenning syndrome. Similarly, a case of Renpenning syndrome has been reported in China, which was associated with the *PQBP1*(NM_001032381.2):c.28C>G (p.Arg10Gly) (Jeong et al., 2018; Germanaud et al., 2011; Pan et al., 2025). However, the lack of prenatal information poses a major challenge for the identification of Renpenning Syndrome.

Currently, international reports are available on Renpenning syndrome in adults with different physical features, making it easy to distinguish and diagnose. However, there is no information is available, and intelligence cannot be determined before delivery. Expanded noninvasive prenatal testing (NIPT-Plus) showed high sensitivity in detecting chromosomal aneuploidy and copy number variations (CNVs) exceeding 5M fragments. Nevertheless, its ability is limited to predefined CNV detection and is prone to false negatives and positives (Chen et al., 2025). NIPT for monogenic diseases is limited to specific genes, and WES is not a routine prenatal test without specific indications. Therefore, only relying on conventional chromosome screening may not be able to detect and diagnose genetic diseases such as Renpenning syndrome in time. The use of ultrasound imaging is essential for identifying fetal structural abnormalities during pregnancy. However, there is no international report on the prenatal ultrasound manifestations of Renpenning syndrome.

The first reported case of prenatal diagnosis of Renpenning Syndrome emphasized the importance of systematic screening during pregnancy.

## Case report

A 35-year-old female, healthy and without a family history of genetic diseases, experienced two abnormal pregnancies. In the first pregnancy, the nuchal translucency (NT) measured 5.5 mm. The decision to terminate the pregnancy before 28 weeks was based on parental choice without chromosome screening. The fetus was male. The patient visited our prenatal diagnosis center for the second pregnancy. Systematic fetal screening was performed in the first trimester of pregnancy. TORCH (Toxoplasma/Other Agents/Rubella Virus/Cytomegalovirus/Herpes Simplex Virus) screening was negative. At 12 weeks, the patient received NIPT, which showed a low-risk outcome. Ultrasound examination showed an NT of 5 mm (HP:0010880) and nasal bones abnormalities (HP:0010937). At 13 weeks, chorionic villus sampling was performed, followed by trio-whole exome sequencing (Trio-WES) using samples of the parents and chorionic villus for family verification. WES was carried out using the Agilent’s SureSelect Human All Exome V6 Capture Kit to prepare libraries, followed by high-throughput sequencing based on the Illumina NovaSeq 6000 platform to obtain high-quality exome data. GRCh37/hg19 was used as the reference genome. CNV was analyzed using the GermlineCNVCaller module in the Genome Analysis Toolkit (GATK). This tool efficiently detected CNV events in WES data applying the sequencing depth information and the output of DetermineGermlineContigPloidy. Trio-WES analysis revealed a 666-bp gene deletion (chrX:48755195-49760422) in the fetus, including *TIMM17B*(NM_001167947.2) and *PQBP1*(NM_001032381.2), inherited from the mother, and no related gene variants or copy number variations were detected in the father. The parents chose to terminate the pregnancy and the fetus was male. According to the 2019 ClimGen/ACMG CNV interpretation guidelines, the pathogenicity classification was considered to be likely pathogenic.

**FIGURE 1:**
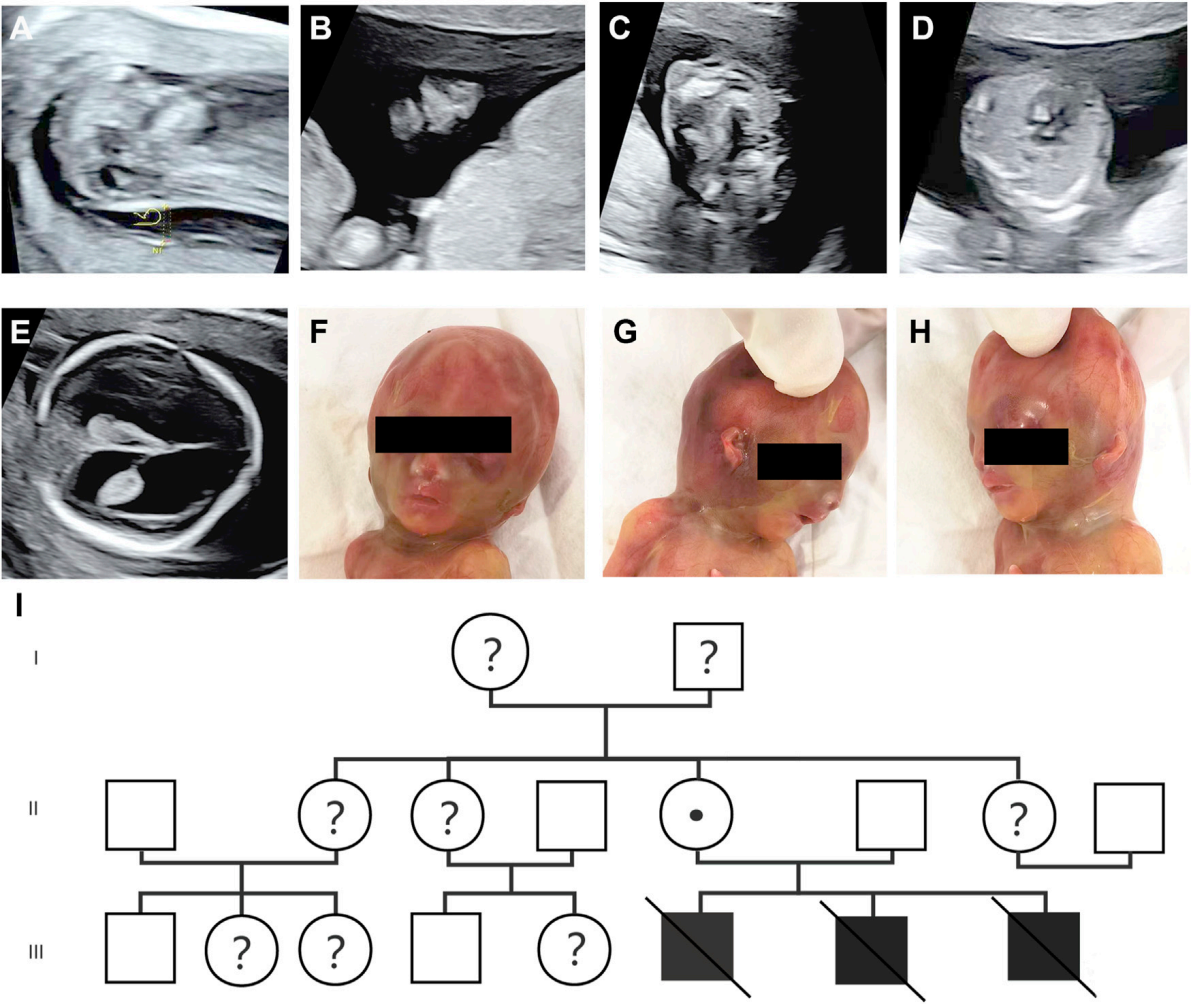
Prenatal ultrasound scan and fetal phenotype after delivery. **(A–C)** Prenatal ultrasound at 12+1 weeks of gestation showed thickened NT (6.5 mm) **(A)**, nasal bone abnormalities **(B)**, and cleft palate **(C)**; **(D,E)** Prenatal ultrasound at 16+1 weeks of gestation showing VSD **(D)**, and mildly enlarged bilateral lateral ventricles **(E)**; **(F–H)** the image of fetus after delivery with a sunken nose and cup-shaped ears; **(I)** the lineage.

During the patient’s third pregnancy, NIPT was performed at 11 weeks, showing a low-risk outcome. Subsequently, an ultrasound scan at 12 weeks showed increased NT (6.5 mm) (HP:0010880), nasal bone abnormalities (HP:0010937) and cleft palate (HP:0000175) at 12+1 weeks (Figures 1A–C). According to medical recommendations, chorionic villus sampling was performed, and MLPA analysis confirmed a 666 BP gene deletion (chrX:48755195-49760422) in the fetus inherited from the mother. An ultrasound scan at 16 weeks revealed the presence of ventricular septal defect (VSD, HP:0001629), and subsequently mild enlargement of bilateral lateral ventricles was observed at 17 weeks (Figures 1D,E). By applying PVS1 and PM2 criteria, prenatal ultrasound could help detect potentially pathogenic *PQBP1* deletions associated with Renpenning syndrome. The pathogenicity level was classified as likely pathogenic according to the 2019 ClimGen/ACMG CNV interpretation guidelines. At 17 weeks of termination of pregnancy, the fetus showed obvious facial features, such as a depressed nose and cup-shaped ears (Figures 1F–H), indicating that the pathogenic *PQBP1* deletion met the criteria PVS1, PM2, and PP4. Based on the results of the ultrasound scan, facial features and genetic testing, the pathogenicity classification was consistent with the 2019 ClimGen/ACMG CNV interpretation guidelines and is pathogenic. Despite the recommendation, the couple refused further pathological autopsy. The pedigree of the patient was shown in Figure 1I, and the schedule of pregnancy information was detailed in Table 1.

**TABLE 1:** The timeline of incorporating information on pregnancy.

Number of pregnancies	Maternal age (years)	Fetal gestational age	Fetal ultrasound	Intervention	Pregnancy outcome	Fetal sex
1	33	12 + 3	NT = 5.5 mm (HP:0010880)	No	Termination of pregnancy	Male
2	34	12 + 3	NT = 5 mm (HP:0010880)	Chorionic villus sampling was performed, followed by trioWES, revealing a 666 bp genetic deletion (chrX:48755195-49760422) inherited from the mother	Termination of pregnancy	Male
3	35	12 + 1	NT = 6.5 mm (HP:0010880), nasal bone abnormality (HP:0010937)	Performing chorionic villus sampling for MLPA analysis	-	-
		16	NT = 6.5 mm (HP:0010880), nasal bone abnormality (HP:0010937), VSD (HP:0001629)	A 666 bp genetic deletion (chrX:48755195-49760422) inherited from the mother	-	-
		17	NT = 6.5 mm (HP:0010880), nasal bone abnormality (HP:0010937), VSD (HP:0001629), mildly enlarged bilateral lateral ventricles (HP:0006956)		Termination of pregnancy, the fetus exhibited distinct facial characteristics such as a sunken nose and cup-shaped ears	Male

The diagnostic challenges in this case included patient compliance, timely prenatal diagnosis, and the high level of expertise required by prenatal diagnostic specialists, such as timely recognition of abnormal ultrasound findings and selection of appropriate chromosome screening methods. After thorough explanation, it was found that the pregnant woman had a 666 BP gene deletion (chrx:48755195-49760422). Then, the probability of the next male fetus being affected was 50%, and the probability of the next female fetus being normal was 50%. As a result, Preimplantation Genetic Testing for Monogenic disorders (PGT-M) was considered. It was recommended to carry out systematic screening in the early stage of the next pregnancy and chromosome screening of chorionic villi at about 12 weeks.

## Discussion and conclusion

At present, international reports mainly focus on Renpenning syndrome in adults, which is characterized by distinct physical features that facilitate differentiation and diagnosis. Most variants in *PQBP1* associated with Renpenning syndrome are frameshift variants in the PRD (POU-specific domain) and CTD (C-terminal domain) regions, leading to premature termination (Kalscheuer et al., 2003; Stevenson et al., 2005). Some known *PQBP1* variants, such as c.727 C>T (p.Arg243Trp) and c.459_462del (p.Arg153SerfsTer41), involve heterozygous variants, deletion frameshift nonsense variants, point missense variants, and duplication variants within the *PQBP1* gene (Pan et al., 2025; Lopez-Martín et al., 2022; Fan et al., 2025). The pathogenesis of Renpenning syndrome involves the regulation of various cellular processes, such as neural progenitor cell proliferation, neural projection, synaptic growth, neuronal survival, and cognitive function, through mRNA transcription and splicing-dependent or -independent mechanisms (Cheng et al., 2023). Moreover, the literature suggests that it is associated with a unique DNA methylation profile (Haghshenas et al., 2023). Renpenning syndrome has been observed only in males, and female carriers are usually asymptomatic. Nevertheless, one study showed that a significant proportion of female carriers in Renpenning syndrome families exhibited skewed X-chromosome inactivation, which complicated the presentation of the syndrome in carriers (Cho et al., 2020).

*TIMM17B* is a gene located on the X chromosome that encodes a translocase present on the inner mitochondrial membrane in humans. It is the only gene in Xp11.23 that shows structural conservation and functional homology on autosomes. This gene encodes a multipass transmembrane protein, which is an important component of the mitochondrial translocase TIM23 complex and is responsible for promoting the transport of proteins containing transport peptides across the inner mitochondrial membrane (Bauer et al., 1999). High expression of *TIMM17B* has been identified as a potential diagnostic and prognostic marker for breast cancer (Mingting et al., 2023). Furthermore, a recent study comprehensively analyzed the genetics of ChrX involvement in autoimmune liver disease and identified a novel genome-wide significant locus associated with primary biliary cholangitis, especially rs7059064. This locus contains seven genes: *TIMM17B*(NM_001167947.2)*, PQBP1*(NM_001032381.2)*, PIM2*(NM_006875.4)*, SLC35A2*(NM_006875.4)*, OTUD5*(NM_006875.4)*, KCND1*(NM_006875.4), and *GRIPAP1*(NM_006875.4), as well as a super-enhancer (GH0XJ048933 in *OTUD5*) that regulates all of these genes (Asselta et al., 2021). Of note, a 666bp deletion (chrX:48755195-49760422) involving *TIMM17B*(NM_001167947.2) and *PQBP1*(NM_001032381.2) was found in this case, which raised doubts about its relevance to primary biliary cholangitis, especially that no biliary abnormalities were detected during ultrasound examination. Although disorders associated with *TIMM17B-*related genes have rarely been reported, the observed fetal phenotype does not definitively exclude a potential influence of *TIMM17B* deficiency, independent of the syndrome caused by *PQBP1* variants.

In this case, a pregnant woman presented with three pregnancies, showing thickened nuchal translucency and nasal bone abnormalities. In addition, prenatal ultrasound revealed VSD (HP:0001629), suggesting cardiac impact. The integration of ultrasound findings, facial features, and genetic testing led to the diagnosis of Renpenning syndrome in the fetus. This case highlighted the importance of early systematic fetal screening. However, there are some limitations to consider. Renpenning syndrome caused by *PQBP1* variants exhibits considerable individual variation. In this case, the fetus had a 666-bp deletion, resulting in a complete loss of *PQBP1*. It is unclear whether other *PQBP1* variants, such as deletion frameshift nonsense variants or point missense variants, could manifest prenatal ultrasound abnormalities. Moreover, the effect of *TIMM17B* on fetal ultrasound findings lacks validation of relevant *in vitro* experiments. Finally, diseases such as Down syndrome may show prenatal ultrasound features similar to Renpenning syndrome. Although the risk of NIPT in this case was low, there were false negatives and false positives in NIPT. In addition to Trio-WES, karyotype analysis was required for differential diagnosis during chromosome screening.

Timely detection and diagnosis of chromosomal diseases during pregnancy is a major challenge. Systematic early screening of fetal development plays a crucial role in identifying genetic conditions that may affect fetal growth. It is recommended to closely monitor fetal ultrasound in the case of multiple adverse pregnancies or obvious genetic susceptibility. If abnormalities are observed, such as increased NT or abnormal ultrasound examinations, prenatal diagnosis should be considered. This may involve chromosomal testing, genomic testing, and WES through procedures such as chorionic villus sampling or amniocentesis to prevent missed diagnosis. Early genetic diagnosis could be carried out to appropriate clinical management and counseling, facilitating informed decision-making and timely clinical intervention.

## Data Availability

The original contributions presented in the study are included in the article/Supplementary Material, further inquiries can be directed to the corresponding author.
